# Results after En Bloc Lateral Wall Decompression Surgery with Orbital Fat Resection in 111 Patients with Graves' Orbitopathy

**DOI:** 10.1155/2015/860849

**Published:** 2015-06-28

**Authors:** Nicole Fichter, Rudolf F. Guthoff

**Affiliations:** ^1^Interdisciplinary Center for Graves' Orbitopathy, Admedico Augenzentrum, Fährweg 10, 4600 Olten, Switzerland; ^2^Department of Ophthalmology, University of Rostock, Doberaner Strasse 140, 18057 Rostock, Germany

## Abstract

*Purpose*. To evaluate the effect of en bloc lateral wall decompression with additional orbital fat resection in terms of exophthalmos reduction and complications. *Methods*. A retrospective, noncomparative case series study from 1999 to 2011 (chart review) in Graves' orbitopathy (GO) patients. The standardized surgical technique involved removal of the lateral orbital wall including the orbital rim via a lid crease approach combined with additional orbital fat resection. Exophthalmos, diplopia, retrobulbar pressure sensation, and complications were analyzed pre- and postoperatively. *Results*. A total of 111 patients (164 orbits) with follow-up >3 months were analysed. Mean exophthalmos reduction was 3.05mm and preoperative orbital pressure sensation resolved or improved in all patients. Visual acuity improved significantly in patients undergoing surgery for rehabilitative or vision threatening purposes. Preoperative diplopia improved in 10 patients (9.0%) but worsened in 5 patients (4.5%), necessitating surgical correction in 3 patients. There were no significant complications; however, one patient had slight hollowing of the temporalis muscle around the scar that did not necessitate revision, and another patient with a circumscribed retraction of the scar itself underwent surgical correction. *Conclusions*. The study confirms the efficiency of en bloc lateral wall decompression in GO in a large series of patients, highlighting the low risk of disturbance of binocular functions and of cosmetic blemish in the temporal midface region.

## 1. Introduction 

Orbital decompression surgery in Graves' orbitopathy (GO) represents an established treatment for rehabilitative exophthalmos reduction and for restoration of visual function in dysthyroid optic neuropathy (DON). A vast number of surgical techniques with regard to orbital decompression are described in the literature. Each orbital surgeon usually favors a particular technique either tailored to the individual patient's needs or the surgeon's preference. The problem in comparing different techniques for orbital decompression is the lack of randomized controlled trials (RCTs). Boboridis and Bunce [1] systematically reviewed the data from all RCTs addressing the issue of orbital decompression surgery. After searching electronic databases, oculoplastic surgery textbooks, conference proceedings, and personal communications from researchers, they found just one RCT comparing different decompression techniques [2] and a second trial comparing medical versus surgical decompression [3]. Furthermore, analysis of data after decompression surgery or comparison of results from different studies is often hampered by the pool of patients, which is highly heterogeneous in terms of both preoperative clinical characteristics and methodological backgrounds and outcome measures.

Hence, our knowledge concerning surgical treatment of disfiguring exophthalmos or compressive neuropathy in GO patients frequently derives from case series involving small patient numbers. Overall there seems to be a trend in favor of lateral wall decompression surgery, as advocated by different authors with excellent track record [4–8]. This technique, in which the rim of the orbital wall is usually preserved or repositioned at the end of surgery, offers the advantage of effective decompression of the orbit combined with a low complication rate. For more than 15 years we successfully used a modified technique with en bloc resection of the lateral wall, including removal of the orbital rim, in order to better visualize the deep lateral orbit and therefore facilitate deep resection towards the greater wing of the sphenoid. The aim of the present study was to evaluate outcomes with this en bloc technique in our patients over a 12-year period.

## 2. Methods

A retrospective case record analysis of GO patients after complete (en bloc) resection of the lateral orbital wall, including the lateral rim, with additional orbital fat resection was performed. The study was approved by the Ethics Committee of the Medical Faculty of the University of Rostock and all investigations were performed according to the current version of the Helsinki Declaration.

### 2.1. Patients

During the period from June 1999 to June 2011 a single surgeon (RFG) performed a total of 201 lateral wall decompression procedures in 130 patients with GO. Patients with a minimum follow-up of 3 months were considered eligible for analysis, and the charts of 111 patients (87 females, 24 males; 164 orbits) were reviewed in detail. The indication for surgery was disfiguring exophthalmos with or without retrobulbar pressure sensation in inactive eye disease in 146 orbits. An additional subset of 18 orbits was found to have mild DON. In 53 patients in whom bilateral decompression was planned, surgery was usually performed in a two-step procedure with a minimum interval of 4 weeks between the two surgical sessions. The mean age of the patients at the time of surgery was 48.8 ± 11.7 years (range: 24–76 years).

Patients who had a surgical orbital intervention before the first consultation were not included in the study. One patient who needed a further medial wall decompression 4 weeks after lateral wall decompression and therefore within the defined follow-up of at least 3 months was not excluded from the study because this shows the importance of a careful and critical preselection of the patients. In this case the endpoint was before the second intervention.

### 2.2. Pre- and Postoperative Measurements

The results of clinical examination before and after surgery concerning visual acuity, exophthalmometer readings, diplopia (Gorman score), and retrobulbar pressure sensation were extracted from the case records. The last follow-up was either the last consultation in our clinic in those patients without further surgical interventions or the last consultation before the next surgical step in rehabilitative surgery (e.g., extraocular muscle surgery or lid surgery).

Conventional lateral rim-supported Hertel exophthalmometers were not suitable for postoperative exophthalmos assessment because our surgical technique involves complete en bloc resection of the lateral orbital wall, including the orbital rim. Therefore we used the superior and inferior orbital rim-based exophthalmometers developed by Naugle Jr. and Couvillion [9] and the measurements were done by multiple examiners. A comparative study by Cole III et al. [10] has concluded that accuracy was comparable for the Naugle and Hertel instruments, justifying our use of the Naugle exophthalmometer for pre- and postoperative measurements. Extraocular muscle involvement was assessed using the Gorman score for classification of diplopia: 1 = no diplopia, 2 = intermittent diplopia (when tired), 3 = inconstant diplopia (depending on direction of gaze), and 4 = constant diplopia in primary or reading position (with or without prism). Information concerning the presence of retrobulbar pressure sensation before and after surgery was also reviewed and documented in a qualitative manner (yes/no). We diagnosed DON if two or more of the following features were present: visual acuity less than 6/6 (decimals), visual field defects in the automated visual field analyser (Humphrey 24-2), impaired color vision (Lanthony panel-D15: >2 minor errors or >1 major error), pathologic visual evoked responses with a prolongation of latency and/or reduction of amplitude, presence of a relative afferent pupillary defect (RAPD), presence of optic disc swelling, or apical crowding in orbital imaging. Patients were led to orbital decompression surgery if there was no adequate response to the medical pretreatment with systemic steroids or steroids were contraindicated. Reported complications and subsequent need for further (rehabilitative) surgical interventions were analyzed in detail.

### 2.3. Surgical Technique

Surgery was performed under general anesthesia and started with an upper eyelid crease incision in the lateral third of the upper lid crease that was extended down to the zygomatic bone about 2 cm lateral to the orbital rim and blunt exposure of the lateral orbital rim. The temporalis muscle was carefully detached from the temporal surface of the lateral orbital wall, followed by incision of the periosteum along the lateral orbital rim. After preparation of the lateral orbital wall with the periosteal elevator the superior osteotomy was marked above the frontozygomatic suture, and the inferior osteotomy was marked just above the zygomatic arch. The osteotomies were performed with an oscillating saw and the fragment of the lateral orbital wall was outfractured using a hammer and osteotome. The amount of bone resection was increased towards the height of the greater wing of the sphenoid using the bone nibbler and burr down to the level of bone marrow. The landmark for the lower border of resection was the inferior orbital fissure. During this procedure the globe and its soft-tissue contents were protected and carefully pulled nasally with malleable retractors. After removing the deep parts of the lateral wall the periorbit was opened superiorly and inferiorly to the lateral rectus muscle and carefully excised. This procedure is usually accompanied by a variable degree of orbital fat prolapse, which is resected mainly from the inferolateral orbit. Additional fat prolapse can be achieved with only minimal risk of orbital bleeding by exerting slight axial pressure on the globe. The amount of fat resection in our series was 2.0 ± 1.0 mL.

Finally a suction drainage system was placed into the fossa temporalis and the wound incision was accurately closed in layers. A temporary compression bandage was used for the first 24 hours in order to maintain axial retropositioning of the globe in the newly created space. This required frequent checks of visual functions and pupillary reflexes. The volume of blood in the suction balloon was monitored every 6 hours in order to be aware of possible postoperative orbital bleeding.

### 2.4. Statistical Analysis

Spearman's rho coefficient was calculated for nonparametric correlations, and *P* values were analyzed using the Wilcoxon nonparametric test. *P* values < 0.05 were accepted as statistically significant, with *P* < 0.01 being regarded as highly significant. Calculations were done using SPSS version 15 for Windows (SPSS Inc., Chicago, IL, USA).

## 3. Results

Mean follow-up was 16.4 ± 20.4 months (range: 3 months to 10.5 years; median: 8 months).

### 3.1. Exophthalmos

**Figure 1:**
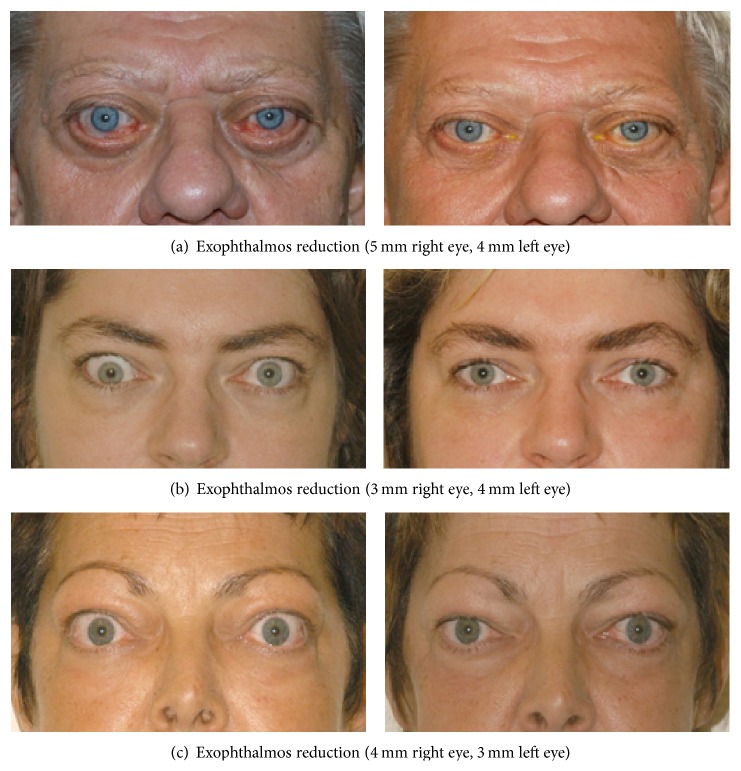
Exemplary cases after bilateral orbital decompression: (a) 69-year-old male patient before and 6 months after surgery. (b) 35-year-old female patient before and 2 years after additional squint surgery. (c) 55-year-old female patient before and 6 months after surgery.

**Figure 2:**
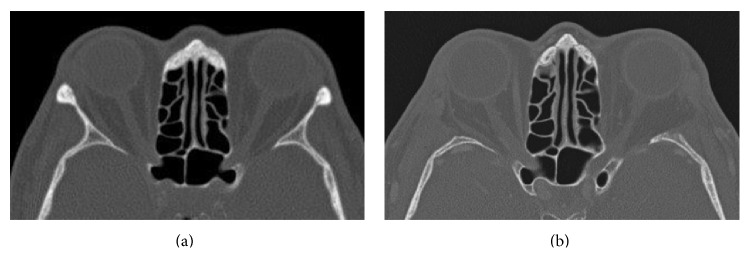
Preoperative (a) and postoperative (b) axial CT scan of the orbit. Note the missing lateral orbital wall after bony decompression without repositioning of the orbital rim.

Mean exophthalmos improved significantly from 21.40 ± 2.3 mm preoperatively to 18.32 ± 2.6 mm postoperatively (*P* < 0.001), resulting in a mean exophthalmos reduction of 3.05 ± 1.45 mm. A moderate correlation was noted between exophthalmos reduction and preoperative exophthalmos. The greater the degree of exophthalmos before surgery, the greater the reduction achieved (*r* = 0.431; *P* < 0.001). For some exemplary pre/postoperative photographs and axial CT scans see Figures 1 and 2.

### 3.2. Visual Acuity

Mean visual acuity (VA) in patients being operated for rehabilitative purposes was 0.91 ± 0.22 preoperatively, increasing to 0.93 ± 0.23 postoperatively (*P* = 0.03). Those patients undergoing surgery because of suspected mild DON initially presented with a mean VA of 0.73 ± 0.21 that increased significantly to 0.83 ± 0.22 (*P* = 0.02) after decompression surgery. Interestingly, the increase was significantly greater in patients with preoperative signs of DON compared to patients without preoperative suspicion of DON (*P* = 0.015).

The DON group included one 52-year-old euthyroid female smoker with inadequate recovery and slowly progressing visual impairment postoperatively, necessitating an additional medial wall decompression. Furthermore one female nonsmoking, euthyroid patient who was treated before for DON on the contralateral eye had no clinical evidence of DON at the time of surgery. She required an additional medial wall decompression 4 weeks after lateral wall decompression because of new-onset visual loss, visual field defects, and prolonged latency in visual evoked responses due to a compressive optic neuropathy with increased extraocular muscle volume. The patient received intravenous high-dose steroids and immediately underwent additional medial wall decompression. Both patients showed a rapid restoration of visual acuity within one week after additional endonasal medial wall decompression surgery.

### 3.3. Retrobulbar Pressure Sensation

A disturbing retrobulbar pressure sensation was reported in 98 out of 164 orbits preoperatively; for 9 orbits the chart records contained no information concerning this symptom. Postoperatively, retrobulbar pressure sensation was reported to be completely resolved in 71 orbits and markedly improved in 3 orbits. No information on this aspect was found in the chart records for 24 orbits.

### 3.4. Strabismus

Preoperatively about one-third of our patients presented without diplopia (Gorman score: 1). Postoperatively, overall distribution within the four Gorman categories showed a slight improvement, that is, a shift to a lower Gorman score, although not statistically significant (Table 1).

Postoperatively, unchanged Gorman scores were observed in 88 patients (87.1%, 135 orbits), diplopia improved in 10 patients (9.0%, 14 orbits), but new-onset diplopia was also noted in 5 patients (4.5%, 6 orbits) (Tables 2(a) and 2(b)). Detailed analysis of patients with an improved diplopia score revealed that preexisting vertical squint improved in 5 of the 10 patients and horizontal squint improved in 3 patients, while 2 patients improved in the sense of resolved intermittent diplopia.

One patient (listed as number 4 in Table 2(b)) moved from “no diplopia” to “inconstant diplopia” 2 months after surgery because of impaired elevation presented with signs of recurrent GO activity in conjunction with recurrent hyperthyroid function and a persisting smoking habit. While worsening of extraocular muscle function was probably not the result of decompression surgery, correction was subsequently done by inferior rectus recession. Two other patients showed deterioration of vertical motility. One patient had a satisfactory field of binocular single vision without impairment of routine daily activities and did not require any further treatment. The deterioration in the second patient who moved from Gorman 1 to Gorman 4 could not be accounted for from the chart record (no intraoperative anomalies; inactive eye disease 5 years after orbital irradiation prior to surgery). After inferior muscle recession a satisfactory field of binocular single vision was obtained.

Worsening of horizontal motility occurred in two patients (adduction in one patient and abduction in the other) with diplopia at the extremes of lateral gaze. The patient with worsened adduction required surgical correction in order to increase the field of binocular single vision. Altogether 5 out of 111 patients (4.5%) showed worsening of extraocular muscle motility presumably due to lateral wall decompression; surgical correction was required in 3 of these patients (2.7%), resulting in a favorable outcome in all of them.

None of the DON patients showed either worsening or improvement of diplopia score.

### 3.5. Additional Surgical Interventions

**Figure 3:**
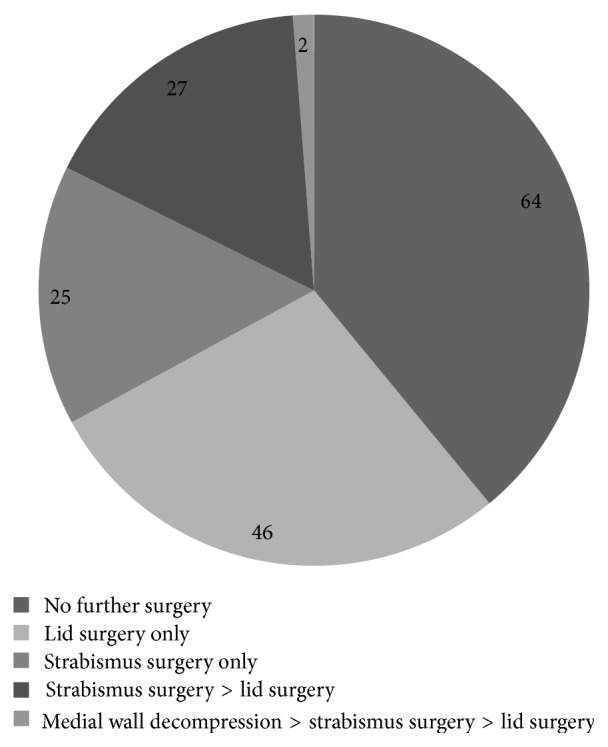
Additional surgical interventions. One or more additional surgical interventions were required in fewer than two-thirds of the orbits after lateral wall decompression. Strabismus surgery was performed in 54 orbits (32.9%) of 43 patients, lid surgery in 75 orbits (45.7%) of 59 patients, and additional medial wall decompression in 2 orbits (1.2%) of two patients.

No further surgical intervention was needed in 64 of the 164 orbits. The remaining 100 orbits (60.9%) required one or more additional surgical procedures (Figure 3). In detail, lid surgery included 58 upper lid lengthening procedures, 16 lower lid lengthening procedures, 10 upper/lower lid blepharoplasties, 4 lateral tarsal sling procedures, 3 lateral tarsorrhaphies, 1 ptosis repair, and 4 wound revisions. Some of the interventions were performed during the same surgical session. Strabismus surgery included 62 rectus muscle recessions (inferior 33, medial 23, lateral 5, and superior 1), 3 Faden procedures (lateral rectus muscle), 1 lateral rectus resection, and 1 inferior oblique muscle recession. Again, some of the procedures were done during the same surgical session.

Further decompression surgery with additional removal of the medial orbital wall was required in two orbits, as mentioned previously.

### 3.6. Complications

Complications concerning extraocular muscle motility and visual acuity are mentioned in the preceding section. Further complications related to wound healing, scar formation, infection, hyperesthesia, and oscillopsia.

**Figure 4:**
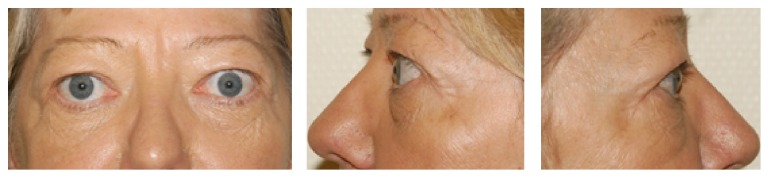
Postoperative hollowing of the temporal fossa region around the scar without any need for treatment (6 months postop).

Visible scar formation manifesting as a retracted scar necessitated revision in one patient. Furthermore one female patient undergoing bilateral decompression experienced a slight hollowing of the temporal fossa region that did not require any further intervention (Figure 4). Oscillopsia when chewing was noticed by one patient but did not affect her related quality of life. Expulsive orbital bleeding shortly after wound closure occurred in one patient; immediate revision with hemostasis led to complete restitution without any functional deficits. An overview of these cases is presented in Table 3.

## 4. Discussion

To our knowledge this study represents the largest retrospective case series after en bloc resection of the lateral orbital wall in conjunction with orbital fat resection. The first results regarding this technique were published in 1966 by Long and Ellis [11] who described a series of 45 decompression procedures. In 1989 Leone Jr. et al. [12] combined this technique with an additional medial wall decompression using Sewall's external approach. In 1991 Matton [13] presented data on 56 decompression procedures in 29 patients following en bloc resection of the lateral wall and lateral floor. Exophthalmos improved in all patients, complications encompassed postoperative bleeding and hypesthesia of the infraorbital nerve, and there was no flattening of the malar contour. Most recently, describing their results after two-wall inferolateral decompression in 44 patients, Schaaf et al. [14] reported an average exophthalmos reduction of 3.8 mm, with one patient developing new-onset diplopia after surgery that required surgical correction.

In the present study we found an average exophthalmos reduction of 3.05 mm, ranging up to 7 mm, after en bloc lateral wall decompression with orbital fat resection. These results are in accordance with our data in an earlier and smaller patient series [15]. Either data do not clarify the extent to which orbital fat resection contributed to exophthalmos reduction, but a review of the literature suggests an additional effect on proptosis reduction to be assumed [16–18]. To achieve this goal our practise is to resect a moderate amount of intraconal fat mainly from the inferolateral orbit as this location is associated with the lowest risk of injury to vulnerable orbital structures [19, 20]. Mourits et al. from the EUGOGO Working Group found a mean exophthalmos reduction after two-wall decompression of 4.3 mm independent of the surgical approach [16]. Thus, exophthalmos reduction after single wall decompression combined with moderate orbital fat resection appears to be slightly less effective than a two-wall decompression technique. However, Ünal et al. [18] postulated that orbital fat resection allows an additional wall to be spared from decompression, thereby reducing the risk of postoperative diplopia. These findings underline the ongoing controversy concerning the role of orbital fat resection in orbital decompression surgery. Additional factors potentially influencing the amount of orbital volume expansion relate to the anatomical variability of the bony orbit [21], variations in the fat-to-muscle volume ratio, the elastic capacity/amount of fibrosis of the orbital contents that determines the ability to fill the newly created space, the orbital opening angle between medial and lateral walls, and the axial lengths of orbit and globe and their relation to each other [22, 23]. Interestingly, orbital irradiation has been shown not to influence the outcome after orbital decompression surgery [24]. But from our personal experience and considering the pathophysiological processes and morphological changes of the orbital soft tissues after irradiation, these results need to be clarified by further studies.

In our study lateral wall decompression surgery produced significant improvement in visual functions in patients with and without DON. The results showed a significantly greater improvement of visual acuity in DON patients compared to patients without preoperative signs of compressive neuropathy, showing that lateral wall decompression can be a sufficient treatment in selected patients with DON. We presume that the improvement of visual functioning in these patients was probably due to a relief of orbital pressure in the sense of an orbital compartment syndrome [25], and in patients without DON this presumably reflected an improvement in ocular surface conditions. Only one of the DON patients (1/18 orbits) was not sufficiently cured and needed further medial wall decompression. This patient was a severe smoker who showed recurrent inflammatory signs in repeated orbital imaging but again without compression of the nerve in the deep orbital apex. Goldberg et al. published their results after decompression of the deep lateral orbit and recommend this technique also for use in DON, but not for cases in the acute inflammatory stage where they advocate additional apical decompression of the medial orbital wall [5]. One comparative study found no difference between lateral and medial walls' decompression in terms of efficacy in treating DON, although lateral wall decompression resulted in greater exophthalmos reduction [26]. Our approach to orbital decompression in DON is to restrict lateral wall decompression to cases with mild DON without signs of optic nerve compression in the deep orbital apex. In patients with a crowded orbital apex we prefer a combined lateral and (endonasal) medial wall decompression to sufficiently decompress the optic nerve and to restore visual functions.

In the present study about one-third of our patients were preoperatively unaffected by diplopia in their routine daily activities and this number did not change significantly after surgery. Detailed scrutiny of the data revealed that some patients (9%) had an improved Gorman score, while a smaller number (4.5%) became worse. The latter underwent surgery between 2003 and 2008, indicating that this complication was probably not related to the surgeon's experience. Interestingly, none of the DON patients showed worsening of preexisting diplopia or new-onset diplopia after surgery. The fact that vertical deviation was equally affected as horizontal deviation indicates that the underlying mechanism relates more to the vulnerability of extraocular muscle balance than possible direct damage to the lateral rectus muscle itself. In line with our findings and using the same surgical technique, Schaaf et al. found an improvement in preexisting strabismus in 14% of their patients and new-onset diplopia in 4.7% (one out of 21 patients) [14]. They found a slight worsening of eye motility on average by 2.4° on upgaze and 0.5° on lateral gaze. Though the authors give no detailed information about the orthoptic assessment these postoperative changes in eye motility do not seem to influence binocular functions and therefore do not seem to influence the patients' quality of life. Further investigations are required to clarify the causative mechanism underlying that observation. Nevertheless, the reported incidence rates of induced diplopia or worsening of motility after different other than lateral wall approaches for orbital decompression are generally higher than those with our technique. A literature review reveals worsening of binocular functions in 16–74% after inferomedial decompression [27, 28], in up to 45% after balanced decompression [5, 29], in 2.6–8% after rim-sparing (deep) lateral wall decompression [5, 30, 31], and in 0–5% after en blocresection of the lateral wall [13, 14].

These latter reports reflect our finding of a low risk for worsening or new-onset diplopia after lateral wall decompression.

Some authors have raised concerns about the potential creation of disfiguring hollowing of the temporal fossa after lateral wall decompression and about complications due to periorbital incisions (e.g., retracted scars or lid retraction) [32] or increased postoperative morbidity as a result of orbital rim removal [8]. Sasim et al. reported temporal bossing in 3 out of 46 patients after coronal 3-wall decompression involving a rim-sparing lateral wall resection that required surgical correction in one of the patients [33]. Bailey described temporalis muscle atrophy after lateral wall decompression via a swinging eyelid approach in 1 out of 55 patients though the lateral rim was repositioned at the end of surgery [34].

In our study one patient had slight hollowing of the temporalis region around the scar that did not require any intervention and was probably due to circumscribed atrophy of the temporalis muscle, such as is known also to occur with the above-mentioned procedures, where the lateral rim was preserved or reconstructed. Hence, the postoperative phenomenon of temporalis wasting does not seem to be caused by the removal of the lateral rim itself rather than by intraoperative temporalis muscle trauma or scarring of the periorbital incision, respectively. The same applies to our patient with a retracted scar and the lady who reported oscillopsia when chewing; both phenomena were probably caused by deep cicatricial adhesions. While the circumscribed retraction of the scar was sufficiently treated by scar revision, mild oscillopsia when chewing remained until the last follow-up at 1 year after surgery but did not affect the patients quality of life. This coincides with a recently published retrospective study which showed the incidence of postoperative oscillopsia after rim-sparing lateral wall decompression to be surprisingly high (35%), but showed it to resolve or improve spontaneously in all but one out of 34 affected patients within 2 years [35].

In summary, these fortunately rare observations underline the importance of an appropriate surgical technique when detaching the temporalis muscle and of a precisely layered wound closure. In addition to our observations in this retrospective study on postoperative temporalis fossa appearance we found no significant influence of the lateral rim removal technique on canthus formation and stability in a prospective study published recently [36]. Furthermore none of the patients ever reported any kind of eye injury after orbital trauma though this could be possible in theory considering the surgical technique removing the lateral orbital rim.

One way to completely avoid these potential problems would be to perform a lateral wall decompression ab interno [31], though this is offset according to some authors by the disadvantage of a more difficult access to the deep lateral orbit with a higher risk for severe complications like CSF leaks and lesser exophthalmos reduction [8, 37, 38]. On the other hand, Rocchi et al. from the Sellari-Franceschini working group [39] who published their results after rim-sparing lateral wall decompression via the internal approach found comparable results for exophthalmos reduction with a negligible risk for new-onset diplopia in primary gaze if patients were free of diplopia preoperatively, while 26.1% of the patients with preoperative inconstant diplopia developed constant diplopia in primary gaze position.

In conclusion, en bloc resection of the lateral orbital wall offers the advantage of good visualization of the surgical field that facilitates resection of the deep lateral wall with a lower risk for severe complications like CSF leaks. Though the limitation of the study is its retrospective, noncontrolled, and noncomparative design the analysis of a huge number of patients supports the recent trend towards lateral wall decompression: the technique has proved to be effective and safe in terms of exophthalmos reduction, postoperative motility disturbances, overall morbidity, and lowest incidence of induced diplopia compared with other orbital decompression techniques. In our opinion, repositioning of the orbital rim does not seem to be necessary with regard to scar retraction and circumscribed hollowing of the temporalis fossa. Concerning exophthalmos reduction Rocchi et al. found comparable results to our technique with a modified internal approach to the lateral orbital wall sparing the anterior orbital rim.

Lateral wall decompression with orbital fat resection is our preferred first choice in patients without disturbance of binocular functions and where moderate exophthalmos reduction is required. Additional medial wall decompression is reserved for patients in whom a lateral wall decompression with or without fat resection might not be sufficient, that is, patients with thickening of the extraocular muscles predominantly in the orbital apex that places the patient at risk to optic nerve compression or in patients where more than 3 mm exophthalmos reduction is needed. The surgical approach therefore has to be customized for each patient according to individual characteristics and the clinical situation.

It therefore seems reasonable to compare our technique with Stellaris' ab interno technique in randomized controlled trials.

## Figures and Tables

**Table 1:** Pre- and postoperative results of diplopia analysis (Gorman score (GS): 1–4). In patients undergoing bilateral decompression surgery postoperative analysis after the first surgical intervention was performed before surgery on the fellow eye.

	GS 1 (no diplopia)	GS 2 (intermittent, diplopia when tired)	GS 3 (inconstant, gaze-dependent diplopia)	GS 4 (constant, diplopia in primary or reading position)
Preoperative (total of 158 orbits)	58 (37.4%)	10	55	35
Postoperative (total of 155 orbits)	61 (39.4%)	9	51	34

**(a):** 

Patient number	Gorman score before surgery	Gorman score after surgery	Subsequent strabismus surgery
1a	3	1	None
1b	3	1	None
2a	3	1	None
2b	3	1	None
3	2	1	None
4a	3	2	None, only rare intermittent diplopia
4b	3	2	None, only rare intermittent diplopia
5a	4	3	None, satisfactory field of binocular single vision after surgery of left eye
5b	4	3	Inferior/internal rectus recession
6	2	1	None
7	3	1	None
8	3	1	None
9	3	2	None, only rare intermittent diplopia
10	3	1	None

**(b):** 

Patient number	Gorman score before surgery	Gorman score after surgery	Subsequent strabismus surgery
1	1	3	None
2	1	4	Inferior muscle recession
3a	1	3	Faden procedure (lateral rectus muscle)
3b	1	3	Faden procedure (lateral rectus muscle)
4	1	3, probably not due to surgery	Inferior muscle recession
5	1	3	None

**Table 3:** Complications after lateral wall decompression surgery in 164 orbits.

Complication	Number of patients/orbits	Treatment
Local hyperesthesia	5 patients, 6 orbits	Revision in 1 patient (1 orbit)) → resolved
Wound infection	1 patient, 1 orbit	Revision
Oscillopsia	1 patient, 1 orbit	No intervention required
Sicca	1 patient, 1 orbit	Lubricants
Visible/subjectively bothersome scar formation	4 patients, 6 orbits	Revision in 1 patient (1 orbit)
Temporal hollowing	1 patient, 2 orbits	No intervention
Early postoperative orbital bleeding	1 patient, 1 orbit	Immediate revision/hemostasis
